# Abnormal electrophysiological phenotypes and sleep deficits in a mouse model of Angelman Syndrome

**DOI:** 10.1186/s13229-021-00416-y

**Published:** 2021-02-06

**Authors:** N. A. Copping, J. L. Silverman

**Affiliations:** MIND Institute and Department of Psychiatry and Behavioral Sciences, University of California Davis School of Medicine, Room 1001B, Research II Building 96, 4625 2nd Avenue, Sacramento, CA 95817 USA

**Keywords:** Genetics, Mouse models, Seizures, Sleep, Behavior, Angelman Syndrome, Ube3a, Spindles

## Abstract

**Background:**

Angelman Syndrome (AS) is a rare genetic disorder characterized by impaired communication, motor and balance deficits, intellectual disabilities, recurring seizures and abnormal sleep patterns. The genetic cause of AS is neuronal-specific loss of expression of *UBE3A* (ubiquitin-protein ligase E6-AP), an imprinted gene. Seizure and sleep disorders are highly prevalent (> 80%) in the AS population. The present experiments were designed to identify translational, neurophysiological outcome measures in a model of AS.

**Methods:**

We used the exon-2 deletion mouse (*Ube3a-*del) on a C57BL/6J background to assess seizure, sleep and electrophysiological phenotypes. Seizure susceptibility has been reported in *Ube3a*-del mice with a variety of seizure induction methods. Here, we provoked seizures by a single high-dose injection of 80 mg/kg pentylenetetrazole. Novel experiments included the utilization of wireless telemetry devices to acquire global electroencephalogram (EEG) and neurophysiological data on electrographic seizures, power spectra, light–dark cycles, sleep stages and sleep spindles in *Ube3a*-del and WT mice.

**Results:**

*Ube3a-*del mice exhibited reduced seizure threshold compared to WT. EEG illustrated that *Ube3a*-del mice had increased epileptiform spiking activity and delta power, which corroborates findings from other laboratories and recapitulates clinical reports in AS. This is the first report to use a cortical surface-based recording by a wireless telemetry device over tethered/fixed head-mount depth recordings. Less time in both paradoxical and slow-wave sleep, longer latencies to paradoxical sleep stages and total less sleep time in Ube3a-del mice were observed compared to WT. For the first time, we detected fewer sleep spindles in the AS mouse model.

**Limitations:**

This study was limited to the exon 2 deletion mouse model, and future work will investigate the rat model of AS, containing a complete *Ube3a* deletion and pair EEG with behavior.

**Conclusions:**

Our data enhance rigor and translatability as our study provides important corroboration of previous reports on epileptiform and elevated delta power. For the first time we report neurophysiological phenotypes collected via translational methodology. Furthermore, this is the first report of reduced sleep spindles, a critical marker of memory consolidation during sleep, in an AS model. Our results are useful outcomes for therapeutic testing.

## Introduction

Angelman Syndrome (AS) is a rare (~ 1:15,000) neurodevelopmental disorder (NDD) characterized by impaired expressive communication skills, ataxia, motor and balance deficits, severe intellectual disabilities, recurring seizures and poor sleep [1, 2]. Among the most frequent (> 80%) clinical features of AS are seizures and abnormal encephalogram (EEG) patterns, where seizures often start early in life and are largely resistant to classic anti-epileptic drugs and distinct EEG signatures commonly precede most clinical features and are persistent throughout an AS individual’s lifetime [3, 4]. Seizures occur frequently and typically present across multiple seizure types including, but not limited to, absence, myoclonic and generalized clonic-tonic seizures and, while they are generally diagnosed early in life, they are consistent throughout an individual’s lifetime, contributing to a significantly higher burden of care [5–7]. Abnormal encephalogram (EEG) patterns are widespread in AS and include epileptiform discharges, increased rhythmic delta wave activity and intermittent elevated theta activity [8, 9]. In addition, sleep deficits are also common in AS (20–80%) and are one of the most difficult symptoms to manage, as reported by parents and caretakers [3, 10].

AS results from the loss of expression and function of the ubiquitin-protein ligase E6-AP (*UBE3A*) gene in neuronal cells [11]. *UBE3A* is maternally imprinted and, as such, is only expressed from the maternal allele in neuronal but not glial cells in the brain albeit biallelically expressed in all other cell types throughout the body. *UBE3A* is located on chromosome 15q11.2–13, and its protein product, Ube3a, mainly functions as a ligase responsible for polyubiquitinating chains to substrates, targeting them for degradation by the proteasome [12]. A number of studies indicate a role for Ube3a in synaptic and neural plasticity, which may underlie the imbalance of excitatory/inhibitory homeostasis and contribute to seizure phenotypes and irregularities in sleep [13–15]. Despite a broad understanding of the genetic etiology and some of the basic mechanistic function(s) of Ube3a, there is an unmet need for therapies for individuals with AS.

While we and others have previously described behavioral deficits in rodent models of AS [16, 17] and other neurodevelopmental disorders (NDDs) [18–26], an effort has been made to incorporate in vivo electrophysiology, expanding clinically analogous phenotypes that can be provided as proof of in vivo efficacy. Pursuant to this goal we sought to identify relevant functional phenotypes, including seizures, EEG signature, sleep patterns and sleep spindles using the most rigorously characterized mouse model of AS, with a deletion of *Ube3a* (*Ube3a*-del) inherited from the maternal allele resulting in *Ube3a*^*m−/p*+^ on the C57BL/6 J background generated from breeding Ube3a^m+/p−^ females with Ube3a^m+/p+^ males [11].

Many laboratories have used this AS mouse model of Jiang and Beaduet [11] and reported numerous characteristics that resemble core features of AS including seizure susceptibility, increased epileptiform activity, elevated delta and sleep deficits, though they vary widely on seizure induction methods, background strain and EEG collection techniques [15, 27–33]. In corroboration and extension, we quantified seizure susceptibility in *Ube3a*-del mice on the seizure-resistant C57BL/6J background strain [34]. Additionally, we investigated baseline epileptiform activity and its indication in hyperexcitability metrics. We compared spectral power signatures using our wireless telemetry devices that employed the use of surface electrodes to quantify cortical EEG while allowing free movement of the animal in its home cage. Finally, we extended earlier reports of sleep deficits and, for the first time, quantified sleep spindles in the *Ube3a*-del mouse model on the C57BL/6J background.

## Materials and methods

### Subjects

All animals were housed in a temperature-controlled vivarium maintained on a 12:12-h light–dark cycle. All procedures were approved by the Institutional Animal Care and Use Committee (IACUC) at the University of California Davis and were conducted in accordance with the National Institutes of Health Guide for the Care and Use of Laboratory Animals. All experiments were performed on B6.129S7-*Ube3a*^*tm1Alb*^/J (*Ube3a*) mice obtained from The Jackson Laboratory (Stock number 016590; Bar Harbor, ME, USA) and housed in a 24-h light–dark cycle (7 a.m.–7 p.m.), temperature controlled room and fed a standard diet of Teklad global 18% protein rodent diets (Envigo, Hayward, CA, USA). To maintain the colony, *Ube3a*^*m*+*/p−*^ male mice were paired with C57BL/6J wildtype females resulting in paternal transmission of the mutant allele that is silenced due to imprinting and litters with normal Ube3a expression. To create mice with maternal transmission of the mutant allele, *Ube3a*^*m*+*/p*+^ (WT) male mice are paired with *Ube3a*^*m−/p*+^ females resulting in *Ube3a-*del mice and their WT littermate controls. To identify mice, pups were labeled by paw tattoo on postnatal day 2–3 using non-toxic animal tattoo ink (Ketchum Manufacturing Inc., Brockville, ON, Canada). At postnatal day 2–7, tails of pups were clipped (1–2 mm) for genotyping, following the UC Davis IACUC policy regarding tissue collection. Genotyping was performed with REDExtract-N-Amp (Sigma-Aldrich, St. Louis, MO, USA) using primers Wildtype Forward: TCA ATG ATA GGG AGA TAA AAC A, Common: GAA AAC ACT AAC ATG GAG CTC and Mutant Forward CTT GTG TAG CGC CAA GTG C.

### Design

Three cohorts of mice were used in this study. All subjects were bred in our facility with a deletion of *Ube3a* (*Ube3a*-del) inherited from the maternal allele resulting in *Ube3a*^*m−/p*+^ mice generated from breeding *Ube3a*^*m*+*/p−*^ females with *Ube3a*^*m*+*/p*+^ males, termed *Ube3a*-del hereafter. Cohort 1 consisted of 20 WT and 8 *Ube3a-*del mice (5 *Ube3a-*del/9 WT males and 3 *Ube3a-*del/11 WT females) that were observed for 30 min before and after an administration of pentylenetetrazole (PTZ; 80 mg/kg; i.p.) for behavioral seizure characterization (SKU: P6500, Sigma-Aldrich, St. Louis, MO, USA). EEG data were acquired in Cohorts 2 and 3 where animals were anesthetized and implanted with a wireless telemetry device designed to measure electroencephalogram (EEG) and electromyogram (EMG) in freely moving animals (Data Science International, New Brighton, MN). Cohort 2 consisted of 7 *Ube3a*-del animals (3 males, 4 females) and 3 WT (2 males, 3 females) that were recorded for 24 h before administration of a lethal dose of pentylenetetrazole (80 mg/kg; i.p.) to observe EEG before and after seizure induction. Cohort 3 consisted of 7 *Ube3a*-del mice (4 males, 3 females) and 5 WT (4 males, 1 female) that were recorded for 72 h to collect sleep data. All animals were between 8 and 12 weeks old, and experimenters were blind to genotype. Subjects were implanted with the EEG device 7 days prior to data acquisition then, on day 8, began testing. All EEG recordings were collected in the subject animal’s home cage in a temperature-controlled testing room maintained on a 12:12-h light–dark cycle. All animals were littermates and singly housed after EEG implantation to avoid possible device displacement due to cage-mate interactions.

### PTZ administration

Seizure induction studies were conducted using 80 mg/kg pentylenetetrazole delivered intraperitoneally. Before administration, subjects were observed for 30 min and weighed to determine the appropriate solution volume. For those that were previously implanted, the weight of the implant (4.0 g) was subtracted from their total weight. Dosing was conducted in the morning (10:00–11:00) in a dim (~ 30 lx) holding room. Directly after administration of the convulsant, subjects were placed in a clean, empty cage where subsequent seizure stages were live-scored for 30 min. Seizure stages were scored using latencies to (1) first jerk/Straub’s tail, (2) loss of righting, (3) generalized clonic-tonic seizure, and (4) death. First jerk/Straub’s tail, previously described by Straub et al. (1911), was identified as a tonic dorsal extension of the tail usually accompanied by a jerk or jump of the animal’s entire body. Loss of righting was defined by the absence of both fore- and hindlimb paws from the surface of the cage bottom for > 1 s. Generalized clonic-tonic seizures were identified as loss of righting followed by phases of rigidity and forelimb/hindlimb spasms. Time to each stage was taken in seconds and analyzed across genotype.

### EEG implantation

Wireless EEG transmitters were implanted in anesthetized test animals using continuous isoflurane (2–4%). A 2–3 cm midline incision was made over the skull and trapezius muscles and then expanded to expose the subcutaneous space. Implants were placed in the subcutaneous pocket lateral to the spine to avoid discomfort of the animal and displacement due to movement. Attached to the implant were 4 biopotential leads made of a nickel-cobalt-based alloy insulated in medical-grade silicone, making up two channels that included a signal and reference lead. These leads were threaded toward the cranial part of the incisions for EEG and EMG placement. The periosteum was cleaned from the skull using a sterile cotton-tip applicator and scalpel; then, two 1-mm-diameter burr holes were drilled (1.0 mm anterior and 1.0 mm lateral; − 3.0 mm posterior and 1.0 mm lateral) relative to bregma. This lead placement allowed for measurement of EEG activity across the frontal cortical area. Steel surgical screws were placed in the burr holes, and the biopotential leads were attached by removing the end of the silicone covering and tying the lead to its respective screw. Once in place, the skull screws and lead connections were secured using dental cement. For EMG lead placement, the trapezius muscles of the animal were exposed, and each lead was looped through and sutured to prevent displacement. Finally, the incision was sutured using non-resorbable suture material and the animals were placed in a heated recovery cage where they received carprofen (5 mg/kg; i.p.) directly after surgery and 24 h post-surgery as an analgesic. Subjects were individually caged with ad libitum access to food and water for 1 week before EEG acquisition and monitored daily to ensure proper incision healing and recovery. Each implantation surgery took < 45 min, and no fatalities were observed.

### EEG data acquisition, processing and analysis

After a 1-week recovery from surgical implantation, individually housed mice were assigned to PhysioTel RPC receiver plates that transmitted data from the EEG implants to a computer via the data exchange matrix using Ponemah software (Data Sciences International, St. Paul, MN). EEG and EMG data were collected at a sampling rate of 500 Hz with a 0.1 Hz high-pass and 100 Hz low-pass bandpass filter. Activity, temperature and signal strength were collected at a sampling rate of 200 Hz. Data acquired in Ponemah was read into Python and further processed with a bandpass filter from 0 to 50 Hz to focus on our frequencies of interest.

#### Power spectral density analysis

Spectral analysis was performed in Python using MEG and EEG Analysis and Visualization (MNE) open-source software. Frequency bands were defined as delta 0.5–4 Hz, theta 5–9 Hz, alpha 9–12 Hz, beta 13–30 Hz, and gamma 30–50 Hz. Spectral power was analyzed using the Welch’s method which windows over the signal and averages across spectral samples. For power spectral densities (PSD) investigated in Cohort 2, analysis started 3 h into recording and finished 3 h prior to the end of recording and PTZ administration, resulting in an 18-h sampling window. PSD analysis in Cohort 3 also began 3 h into recording but continued over the three-day recording resulting in a 69-h sampling window. No statistical difference was detected in PSD within genotype between samples; therefore, both cohorts were combined. Total delta power was determined by adding the density data detected in the 0.5–4 Hz frequency range, while total power summed all the power spectral density data in the 0.5–50 Hz frequency range. Relative delta frequencies were calculated by dividing total delta power by total power per animal and averaging across genotype.

#### Spiking analysis

For spiking analysis, baseline EEG data were segmented into 30-s windows where the mean amplitude was calculated per window. Spiking analysis was conducted in data collected in the 24 h prior to PTZ administration in Cohort 2 and all of the data collected in Cohort 3. In a first-pass assessment, potential spike data were demarcated as any point 2.5 standard deviations above or below the mean amplitude of a given window. To determine true spike events, the data were then filtered for peaks which were defined as points where the three data points prior to and following the peak were increasing and decreasing in amplitude, respectively, to the potential peak of interest. If activity was detected during a 30-s window, that data were not included in the spike count to avoid possible movement artifact. Similar to PSD analyses, the first and final 3 h was removed from the spiking data for both cohorts. Spiking activity could not be combined between Cohorts 2 and 3 as the difference in recording time (24 versus 72 h) greatly contributed to the number of spikes detected.

#### Sleep analysis

Sleep in mice was assessed using EEG/EMG signals and automatically binned with Neuroscore software (Data Sciences International, St. Paul, MN) into active wake, wake, slow-wave sleep or paradoxical sleep states. A wake state was characterized by a low-amplitude, high-frequency signal with low-EMG tone, while an active wake state was distinguished by high-EMG tone. Sleep was divided into either a slow-wave sleep state or a paradoxical sleep state. Slow-wave sleep was defined by having a high-amplitude, low-frequency signal with elevated delta power and low-EMG tone, while paradoxical sleep had a low-amplitude, low-frequency signal with elevated theta power and low-EMG tone. EEG data were segmented into 1-s windows, and the sleep stage was determined by Neuroscore. We defined a scoring epoch of 10-s and, if at least 50% of the epoch was predominantly one type of sleep stage, that epoch was marked with the majority stage. If a 50% criterion was not reached, then that epoch was not included in analysis. Sleep/wake stages were evaluated in Cohort 3 for the entirety of the acquisition period as this cohort did not conclude with seizure induction. Mean time in a sleep state was calculated by averaging the time spent in each bout of that state. Sleep latency was defined as the average latency to a sleep state from either active wake or wake. Total sleep time was summed across the entirety of the recording from sleep state bouts.

For sleep parameter analysis across light–dark cycles, the first 24-h time period was sectioned into 2-h time bins starting at 12:00 a.m. (0–2 time of day). Paradoxical sleep and slow-wave sleep were evaluated separately, while active wake and wake stages were combined into awake readouts. Raw counts of awake, paradoxical sleep, slow-wave sleep or artifact were taken every 10 s per 2-h time bin, resulting in 720 possible scores. To determine percent time in each sleep stage, the total count of each sleep stage was divided by the total possible count (720) and multiplied by 100. To further quantify both sleep stage counts and percent sleep time between light–dark cycles, sleep data were combined for the 7–19 time bins and 0–7 and 19–24 time bins. Counts and percent sleep time during the 7–19 time bins were considered the “light cycle,” while those from the 0–7 and 19–24 time bins were the “dark cycle.”

#### Sleep spindle analysis

To identify and analyze sleep spindles, we developed a custom Python script modified from a study designed to validate automated sleep spindle detection [35]. Briefly, a bandpass filter with cutoff frequencies of 10 and 15 Hz was applied to include the mouse spindle peak frequency of 11 Hz [36]. Additionally, a Butterworth filter (3 Hz first stopband, 10 Hz first passband, 15 Hz second passband, 22 Hz second stopband, 24 dB attenuation level) was used to further filter for the frequency bands of interest. Next, the root-mean square (RMS) of the filtered signal was calculated with a 750-ms window to smooth the EEG trace before cubing the entire signal to amplify the signal–noise ratio. To detect spindles, a lower threshold (1.2 × mean-cubed RMS) was used to determine the start and end of a spindle, while an upper threshold (3.5 × mean-cubed RMS) was used to identify the peak of a spindle. Finally, a spindle had to be longer than 0.5 s and less than 10 s for detection. Spindle detection was analyzed for the entirety of the acquisition period of 72 h in Cohort 3.

#### Statistical analysis

**Fig. 1:**
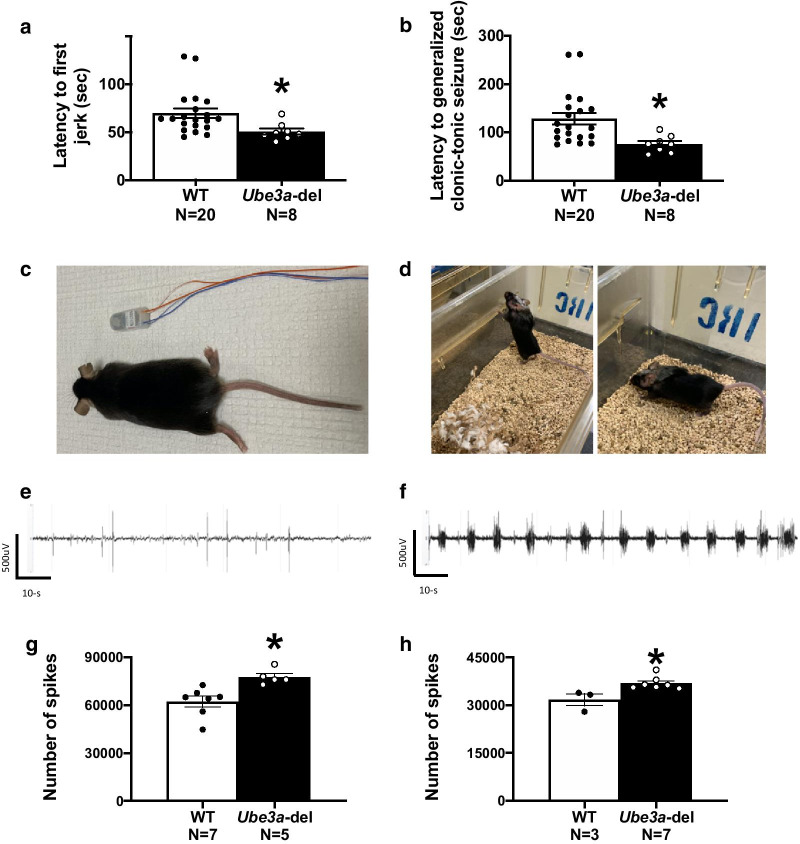
*Ube3a-*del mice exhibited seizure susceptibility and increased spiking compared to WT during baseline EEG recordings. Seizure susceptibility measures were observed for 30 min before and after an i.p. injection of 80 mg/kg PTZ. **a** Reduced latencies to first jerk were observed in the *Ube3a*-del mice compared to WT. **b** Faster onset to generalized tonic–clonic seizure was observed in *Ube3a*-del versus WT. To assess hyperexcitability, **c** mice were implanted with a telemetric device that collected both EEG and EMG data and **d** was small enough to allow for untethered, unrestrained data collection from the home cage of the test animals. **e, f** Representative EEG traces of both WT and *Ube3a-*del animals sampled before convulsant administration. Quantification of spiking activity during baseline data acquisition in mice recorded for **g** 72 h and **h **24 h indicated more spiking events in *Ube3a*-del animals compared to their WT littermate controls. *p < 0.05, Student’s *t *test between genotype

**Fig. 2:**
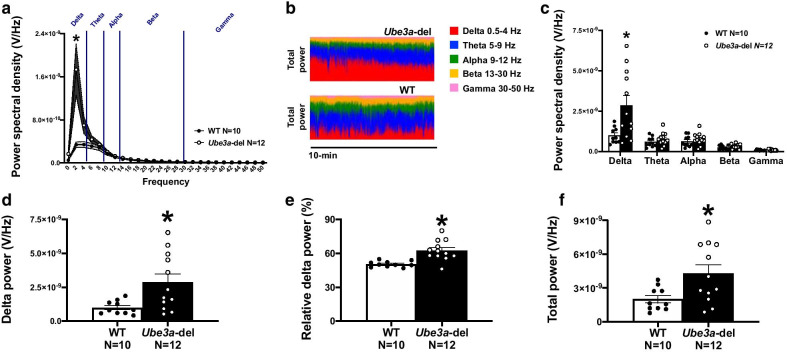
*Ube3a-*del mice exhibited elevated delta power. Surface EEG was collected in mice via a wireless telemetric device and analyzed for spectral power dissimilarities in delta (0.5–4 Hz), theta (5–9 Hz), alpha (9–12 Hz), beta (13–30 Hz) and gamma (30–50 Hz) frequencies during baseline EEG recordings. **a**, **c** Power spectral density collected from *Ube3a-*del mice differed from WT, specifically in the delta frequency range where *Ube3a-*del mice had higher delta power. No significant differences were detected at theta, alpha, beta or gamma frequencies by genotype. **b** Representative 10-min power distributions of *Ube3a-*del and WT mice illustrated the elevated delta phenotype observed in deletion animals. Power spectral analysis was also evaluated by total delta power (summation of power spectral densities across the delta frequency per animal), relative delta power (total delta power of a given animal divided by that animal’s total power) and total power (summation of power spectral densities across all frequencies per animal) between genotype. Quantification of **d** delta power and **e** relative delta power was significantly increased in *Ube3a-*del mice compared to WT littermate controls. **c** Total power was also significantly higher in *Ube3a-*del animals due to their elevated delta power. *p < 0.05, Two-way RM ANOVA comparing genotypes across frequency and Student’s *t *test between genotype

**Fig. 3:**
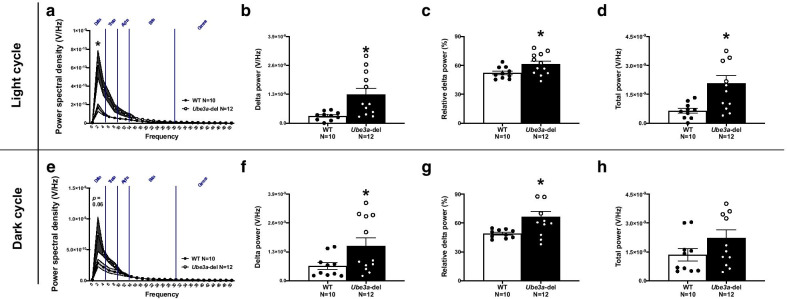
Elevation in delta power is persistent across light and dark cycles in *Ube3a-*del mice. Power spectral densities across all frequency bands were analyzed by light–dark cycles as well as delta power, relative delta power and total power between genotype. **a** Power spectral density collected from *Ube3a-*del mice during the light cycle differed from WT, specifically in the delta frequency range where *Ube3a-*del mice had higher delta power. **e** Trends toward elevated power spectral densities were also observed in *Ube3a-*del mice during the dark cycle. Quantification of **b**, **f** delta power and **c**, **g** relative delta power was significantly increased in *Ube3a-*del mice compared to WT littermate controls across light and dark cycles. **d**, **h** Total power was higher in *Ube3a-*del animals during the light cycle, with trends toward significance during the dark cycle (*p* = 0.125), due to their elevated delta power. *p < 0.05, Two-way RM ANOVA comparing genotypes across frequency and Student’s *t *test between genotype

**Fig. 4:**
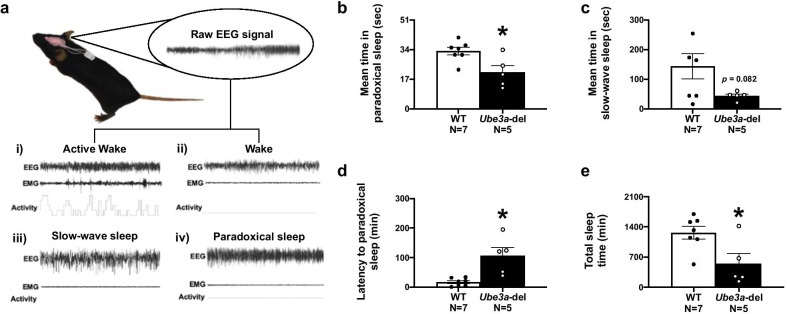
Sleep–wake cycles are altered in *Ube3a-*del mice. **a** A schematic detailing how each sleep state is binned using representative samples. A **(i)** wake state was characterized by a low-amplitude, high-frequency signal with low-EMG tone, while an **(ii)** active wake state was distinguished by high-EMG tone and activity. Sleep was divided into either a slow-wave sleep state or a paradoxical sleep state. **(iii)** Slow-wave sleep was defined by having a high-amplitude, low-frequency signal with elevated delta power and low-EMG tone, while **(iv)** paradoxical sleep had a low-amplitude, low-frequency signal with elevated theta power and low-EMG tone. *Ube3a-*del mice had significantly reduced **b** mean time in paradoxical sleep and **d** increased latencies to paradoxical sleep states, suggesting that deep sleep in deletion mice was not only shorter, but delayed compared to WT littermate controls. Additionally, *Ube3a-*del mice exhibited decreased mean time in **c** slow-wave sleep that when combined with the paradoxical sleep deficits resulted in lower **h** total sleep time compared to WT controls. *p < 0.05, Student’s *t *test between genotype

**Fig. 5:**
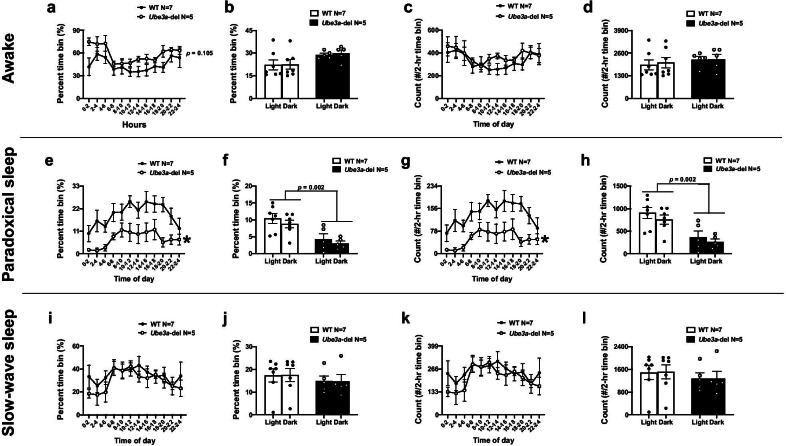
Sleep deficits observed in *Ube3a-*del mice are persistent across light–dark cycles. Sleep parameters were analyzed across light–dark cycles by sectioning the first 24-h time period into 2-h time bins starting at 12:00 a.m. (0–2 time of day). Time in each sleep stage, represented as percent of a 2-h time bin, and bouts of sleep stages, represented as counts per 2-h time bin, were examined. Both wake and active wake stages were combined for awake analysis. Grey boxes highlighting hours 7–19 indicate the dark cycle, while unhighlighted hours ranging from 0 to 7 and 19–24 indicate light cycle. **a** A trend toward increased percent time awake was detected in *Ube3a-*del mice compared to WT littermate controls (*p* = 0.105), **b** that was not specific to either the light or dark cycle. **c** Awake bouts were not significantly different between *Ube3a-*del mice compared to WT, and **d** no significant differences were observed between light and dark cycles. Interestingly, **e** *Ube3a*-del mice exhibited less percent paradoxical sleep time compared to WT controls that was **f** significant across both the light and dark cycles. **g** Likewise, paradoxical sleep bouts were significantly reduced in *Ube3a*-del mice compared to WT littermate controls, again **h** across both light and dark cycles. Both percent time (**i**) and bouts (**k**) of slow-wave sleep were not significantly different between genotypes or across light–dark cycles (**j**, **l**). *p < 0.05, Two-way RM ANOVA comparing genotypes across time bins and mixed model effects between genotype and light–dark cycle

**Fig. 6:**
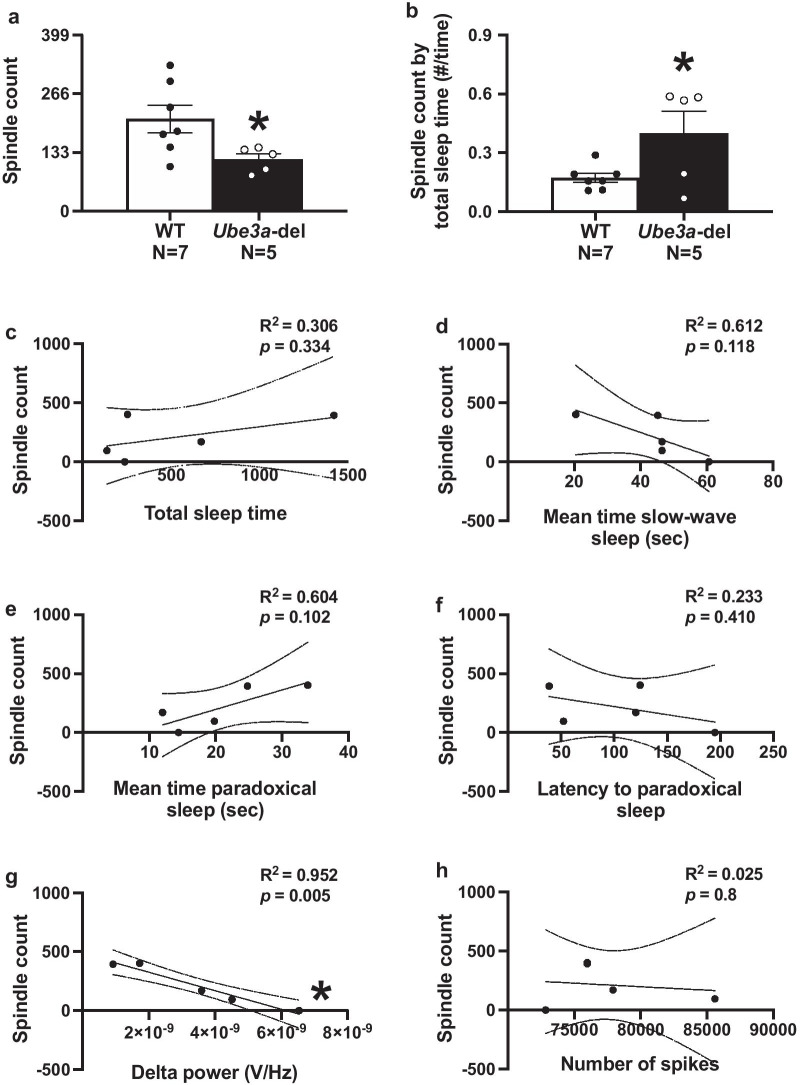
*Ube3a-*del mice exhibited reduced sleep spindle production that was significantly correlated with delta rhythmicity. Sleep spindles were automatically detected using a custom written Python script. **a** *Ube3a-*del mice had significantly less sleep spindles when compared to their wildtype littermate controls. When taking spindles as a fraction of total sleep time, **b** *Ube3a*-del animals had more spindle counts given their reduced sleep time, suggesting increased frequency of spindle counts during their infrequent sleep periods. To further analyze the *Ube3a*-del spindle count by reported sleep deficits **c–f** correlational analyses were run between spindles and total sleep time, mean time in slow-wave sleep, mean time in paradoxical sleep and latency to paradoxical sleep. No significant correlations were detected between spindle count and sleep. Additionally, **f** spindle production was correlated with spiking events to see if elevated spiking in *Ube3a-*del mice could predict spindle count, but no relationship was found. Finally, **g** spindle count was negatively correlated with delta power in *Ube3a-*del mice, where animals with enhanced delta had less spindles than animals with lower delta power. *p < 0.05, Student’s *t *test between genotype and linear regression for correlation across behaviors

All statistical analyses were performed in Prism (version 8, GraphPad Software, San Diego, CA, USA), and data are shown as mean ± standard error. All data sets were tested for outliers using the Rout test with *Q* = 1%. For seizure susceptibility (Fig. 1a, b, Additional file 1: Fig. 1a, b), spiking activity (Fig. 1g, h), power spectral comparisons (Fig. 2d–f), light–dark power spectral comparisons (Fig. 3b–d, f–h), sleep parameters (Fig. 4b–e) and spindles (Fig. 6a, b) Student’s *t* tests were used to test significance and *t*, degrees of freedom and *p* values are reported. Two-way ANOVAs were used to analyze power spectral density differences between genotypes, and the Holm–Sidak multiple comparison post hoc test was used for each frequency band (Figs. 2a, c, 3a, e). Additionally, two-way ANOVAs were used to analyze percent time and counts of sleep stages across time bins and between genotypes (Fig. 5a, c, e, g, i, k). *F*, degrees of freedom and *p* values are reported. Mixed effects models were used to analyze percent time and count of sleep stages between genotypes and light–dark cycles (Fig. 5b, d, f, h, j, l), and Tukey’s multiple comparison post hoc test was used for post hoc analysis. *F*, degrees of freedom and *p* values are reported for mixed effects models, and *p* values are reported for multiple comparisons. Finally, simple linear regression was used for all correlation data (Fig. 6c–f, and Additional file 2: Fig. 2a–j). *F*, degrees of freedom and *p* values are reported in the text, and *R*^2^ values are provided in the figures. All statistics are provided in the text, and “*” indicates *p* < 0.05.

## Results

### Increased seizure susceptibility and spiking activity in *Ube3a-*del mice.

Seizure susceptibility was evaluated in Cohort 1 by latency to (1) first myoclonic jerk and/or Straub’s tail, (2) loss of righting reflex, (3) generalized clonic-tonic seizure and (4) death after administration of PTZ (80 mg/kg; i.p.). A reduction in latency indicated susceptibility, while an increase in latency indicated resistance. *Ube3a-*del animals exhibited seizure susceptibility via reduced latency to first jerk and generalized clonic-tonic seizure (Fig. 1a, b: *t*_(26)_ = 2.287, *p* = 0.031; *t*_(26)_ = 2.627, *p* = 0.014), compared to WT. No differences were detected in loss of righting reflex or death (Additional file 1: Fig. 1a, b: *t*_(26)_ = 1.461, *p* = 0.156; *t*_(26)_ = 1.333, *p* = 0.194). Hyperexcitability and seizure susceptibility were further analyzed by implanting Cohort 2 subjects with a wireless telemetric device that captured continuous EEG (Fig. 1c, d). Epileptiform activity, such as spiking, is sufficient in the detection and diagnosis of epilepsy. As expected, *Ube3a-*del mice in both Cohort 2 and Cohort 3 exhibited increased spiking activity during baseline recording compared to WT controls (Fig. 1g, h: *t*_(10)_ = 3.435, *p* = 0.006; *t*_(8)_ = 3.132, *p* = 0.014).

### Elevated delta power in *Ube3a-*del mice

Rhythmic delta activity is a consistent spectral signature of AS [37, 38] and has been previously reported in mouse models [31, 32], but not via a single-channel electrode placed in the skull via a wireless telemetric system. To test whether our methodology was able to capture EEG signal sensitivity that would also detect increased delta activity, we evaluated spectral power dynamics of WT and *Ube3a-*del mice. When comparing power spectral densities across 0–50 Hz frequencies, *Ube3a-*del mice displayed robust elevated delta power (Fig. 2a: *F*_(1, 20)_ = 6.432, *p* = 0.020; *t*_(20)_ = 2.763, *p* = 0.012 for delta). When densities were binned into delta, theta, alpha, beta and gamma frequency groups, delta power in *Ube3a-*del mice was significantly higher than WT littermate controls (Fig. 2c: *F*_(1, 20)_ = 5.862, *p* = 0.025; *t*_(20)_ = 2.763, *p* = 0.012 for delta).

Delta-specific dynamics were further examined by looking at total delta, relative delta and total power across the entire recording. *Ube3a-*del mice exhibited higher total and relative delta power compared to WT across the entire recording (Fig. 2d, e: *t*_(20)_ = 2.763, *p* = 0.012; *t*_(20)_ = 4.089, *p* = 0.0001). Interestingly, total power was also higher in *Ube3a-*del animals, likely a result of the robust increase in delta (Fig. 2f: *t*_(20)_ = 2.564, *p* = 0.019).

As delta activity is elevated in sleep stages, particularly slow-wave sleep, delta activity was also analyzed across light–dark cycle to control for the possible influence of sleep–wake cycles. When looking at the light cycle, elevated delta activity was detected across all 0–50 Hz power spectral densities (Fig. 3a: *F*_(1, 20)_ = 8.263, *p* = 0.009; *t*_(20)_ = 3.020, *p* = 0.007 for delta). Furthermore, both relative and total delta power were significantly higher in *Ube3a-*del animals as well as total power compared to WT (Fig. 3b–d: *t*_(20)_ = 3.042, *p* = 0.006; *t*_(20)_ = 2.278, *p* = 0.034; *t*_(20)_ = 2.989, *p* = 0.007). Similarly, in the dark cycle, *Ube3a-*del mice displayed elevated delta activity (Fig. 3e: *t*_(20)_ = 2.178, *p* = 0.042 for delta), with trends toward significantly increased PSDs across the 0–50 Hz frequency bins (Fig. 3e: *F*_(1, 20)_ = 4.027, *p* = 0.059). Unsurprisingly, higher total and relative delta power was observed in *Ube3a-*del animals; however, no change in total power was detected (Fig. 3f–h: *t*_(20)_ = 2.191, *p* = 0.041; *t*_(20)_ = 2.823, *p* = 0.011; *t*_(20)_ = 1.60, *p* = 0.125).

### Sleep deficits and abnormal sleep–wake cycles detected in *Ube3a-*del mice

Sleep data were acquired in Cohort 3 animals and subsequently parsed into 4 distinct sleep–wake stages: active wake, wake, slow-wave sleep, and paradoxical sleep. A wake state was characterized by a low-amplitude, high-frequency signal with low-EMG tone, while an active wake state was distinguished by high-EMG tone. Sleep was divided into either a slow-wave sleep state or a paradoxical sleep state. Slow-wave sleep was defined by having a high-amplitude, low-frequency signal with elevated delta power and low-EMG tone, while paradoxical sleep had a low-amplitude, low-frequency signal with elevated theta power and low-EMG tone. *Ube3a-*del mice displayed reduced mean time in paradoxical sleep (Fig. 4b: *t*_(10)_ = 2.91, *p* = 0.016) and took longer to reach paradoxical sleep stages compared to WT (Fig. 4d: *t*_(10)_ = 3.691, *p* = 0.004). Furthermore, trends toward reduced mean time in slow-wave sleep were detected in *Ube3a-*del mice (Fig. 4d: *t*_(10)_ = 1.931, *p* = 0.0823), contributing to a significantly reduced total sleep time compared to WT littermate controls (Fig. 4e: *t*_(10)_ = 2.741, *p* = 0.021).

Sleep parameters were further analyzed by light–dark cycles. The first 24-h time period was sectioned into 2-h time bins starting at 12:00 a.m. (0–2 time of day) where counts of each sleep stage were scored as well as the percent time of a given sleep stage. Both wake and active wake stages were combined for awake analysis. A trend toward increased percent awake time was detected in *Ube3a-*del mice compared to WT littermate controls (Fig. 5a: *F*_(1,10)_ = 3.173, *p* = 0.105) that was consistent across both the light and dark cycles (Fig. 5b: *F*_(1, 6)_ = 4.105, *p* = 0.089). No change in awake counts was observed across time bins or light–dark cycles between genotypes (Fig. 5c: *F*_(1,10)_ = 0.465, *p* = 0.511; Fig. 5d: *F*_(1,6)_ = 465, *p* = 0.521). Interestingly, *Ube3a-*del animals had reduced percent paradoxical sleep time across time bins (Fig. 5e: *F*_(1,10)_ = 12.72, *p* = 0.005) that was observed for both the light and dark cycle compared to WT littermate controls (Fig. 5f: *F*_(1,6)_ = 28.02, *p* = 0.002; WT and *Ube3a-*del by light cycle *p* = 0.009; WT and *Ube3a-*del by dark cycle *p* = 0.014). Expectedly, *Ube3a-*del mice showed decreased paradoxical sleep counts across time bins (Fig. 5g: *F*_(1,10)_ = 12.71, *p* = 0.005) that was reduced in both light and dark cycles compared to WT (Fig. 5h: Genotype effect *F*_(1,6)_ = 28.01, *p* = 0.002; WT and *Ube3a-*del by light cycle *p* = 0.009; WT and *Ube3a-*del by dark cycle *p* = 0.014). Finally, the percent time in slow-wave sleep was not significantly different between genotypes (Fig. 5i: *F*_(1,10)_ = 0.399, *p* = 0.542), or across light–dark cycle (Fig. 5j: *F*_(1,6)_ = 1.310, *p* = 0.296). No significant differences in slow-wave sleep counts were detected across both time bins (Fig. 5k: *F*_(1,10)_ = 0.397, *p* = 0.543) and between light–dark cycles (Fig. 5l: Genotype effect *F*_(1,6)_ = 1.302, *p* = 0.297).

### Sleep spindles are reduced in *Ube3a-*del mice and negatively correlated with the elevated delta phenotype

We wanted to identify, validate and quantify sleep spindles in the *Ube3a-*del mouse, adding another clinically relevant functional phenotype for therapeutic testing. First, a 10–15 Hz bandpass filter was first applied, followed by a Butterworth filter (3 Hz first stopband, 10 Hz first passband, 15 Hz second passband, 22 Hz second stopband, 24 dB attenuation level) for the frequency bands of interest. Next, the root-mean square (RMS) of the filtered signal was calculated with a 750-ms window to smooth the EEG trace before cubing the entire signal to amplify the signal–noise ratio. To detect spindles, a lower threshold (1.2 × mean-cubed RMS) was used to determine the start and end of a spindle, while an upper threshold (3.5 × mean-cubed RMS) was used to identify the peak of a spindle. *Ube3a-*del mice exhibited less sleep spindles total compared to WT littermate controls (Fig. 6a: *t*_(10)_ = 2.357, *p* = 0.04). To control for sleep time we first divided spindle count by total sleep time. Curiously, *Ube3a*-del mice had significantly higher spindle production during their shorter sleep periods compared to WT controls (Fig. 6b: *t*_(10)_ = 2.361, *p* = 0.04). As additional controls for sleep, spindle count was correlated with total sleep time (Fig. 6c: *F*_(1,3)_ = 1.322, *p* = 0.334), mean time in slow-wave sleep (Fig. 6d: *F*_(1,3)_ = 4.723, *p* = 0.118), mean time in paradoxical sleep (Fig. 6e: *F*_(1,3)_ = 5.422, *p* = 0.102) and latency to paradoxical sleep (Fig. 6f: *F*_(1,3)_ = 0.913, *p* = 0.410) in *Ube3a-*del animals. No significant correlations were detected between spindle count and any other sleep metric. Spindle count and spiking count correlations were also analyzed, though no significant correlation was detected (Fig. 6h: *F*_(1,3)_ = 0.077, *p* = 0.800). Interestingly, spindle count was negatively correlated with elevated delta power, where animals with higher delta power had lower spindle counts (Fig. 6g: *F*_(1,3)_ = 59.44, *p* = 0.005). Additional correlation studies showed no significant relationship between spiking, delta and sleep phenotypes (Additional file 2: Fig. 2a–j).

## Discussion

Novel therapies in development for genetic precision medicine for AS that could be “curative” have resulted in (1) unsilencing of paternal Ube3a; (2) molecular reversal of *Ube3a* expression levels; and (3) some degree of functional phenotypic rescue by a wide variety of molecular therapies. These include gene therapy by antisense oligonucleotides (ASO) [39], viral vector delivery [40], and artificial transcription factors (ATFs) [41]. In fact, two ASO compounds are in Phase I clinical trials (GeneTx NCT04259281; Roche NCT04428281). Outcome measures are required to demonstrate the utility of these innovative therapeutic designs as well as to validate other traditional medicinal therapies that may be in the drug discovery pipeline by biotechnology and pharmaceutical companies for AS. Previous research has comprehensively characterized the AS mouse model line of Jiang and Beaduet both behaviorally and biochemically and discovered motor abnormalities [42], learning and memory deficits in fear conditioning [39, 40], strain-dependent seizure susceptibility [15, 32], elevated delta spectral power [31] and abnormal sleep signatures [43]. This study rigorously reproduced findings on hyperexcitability and seizure susceptibility and extended studies using a unique chemoconvulsant mechanism, GABA_B_ antagonist, pentylenetetrazole. Innovation reported herein is the detection of similar effect sizes and phenotypes using a translational approach of acquisition of EEG in the home cage over several days via a skull screw and wireless telemetry over hippocampal depth recordings of local field potentials requiring a head mount and tethered system. Previous work from Sahin and Rotenberg has established this technology as useful for models of NDDs, by their extensive studies in Tuberous Sclerosis Complex and Phelan–McDermid Syndrome models [24, 44]. Both of these genetic NDDs share common phenotypes with AS, including motor difficulties, intellectual disabilities, recurring seizures and sleep difficulties, in addition to diagnoses of autism spectrum disorders (ASD).

It is interesting, although not surprising, that no behavioral seizures were observed in either the mouse or rat model of AS, given it has been recognized by our laboratory and others that mouse models on C57BL6/J backgrounds are resistant to seizures [22, 34]. Since multiple genetic NDDs that are syndromic forms of ASD have high seizure comorbidity, our laboratory adapted to uncovering subthreshold behavioral seizures with wireless, untethered, telemetry implants that acquire the EEG signal. This is the first report to identify with translationally relevant methods, global (non-hippocampal; non-depth local field potentials; freely moving non-tethered) neurophysiology and sleep in AS mice.

In addition to observations of epileptiform and spike trains via EEG in AS subjects, we observed elevated spectral power in the delta frequency band in mice with *Ube3a*^*m−/p*+^ deletions. Our laboratory works closely with patient advocacy groups and gathers observations of clinical AS from leading epileptologists (Thibert, Anderson) providing us numerous deidentified clinical examples for which EEG signatures in individuals with mutations or excess expression of *Ube3a* are unique and distinguishable. For example, Drs. Thibert and Anderson have carefully explained their reports of elevated delta power in AS clinics [31, 32]. Further, we reproduced Sidorov et al. earlier work [45] that highlighted delta power as translational biomarker [31, 33]. This phenotypic reliability paves the pathway to power spectral signatures as therapeutic windows for precision treatment within AS and beyond including other NDDs, such as Dup15q and genetic forms of ASD [46–49].

Sleep is greatly affected in individuals with AS [50–52]. Sleep disturbances reported include reduced overall time sleeping, higher number of nighttime awakenings, and longer onset latencies to falling asleep are the most common [53, 54]. Sleep analysis is highly translational, since a majority of genetic NDDs, including AS, have clinical sleep disruption. This report defined rodent sleep cycles over a 36-h acquisition period. We measured alterations by quantifying time in: (a) wake, defined as high-frequency, low-amplitude signals with without EMG or video activity; (b) active wake, similar to wake but with detected activity; (c) time in slow-wave sleep, defined as low-frequency, high-amplitude signal with elevated delta; (d) time to sleep onset and time spent in paradoxical (~ REM) sleep, characterized by a low-frequency, low-amplitude signal with elevated theta.

We reproduced Ehlen and Philpot’s earlier work [45] that maternal Ube3a loss had a striking effect on the architecture of sleep–wake cycles. As reported, *Ube3a*-del had reduced REM sleep bouts when employing 2-h time bins to establish percent time scores in *Ube3a*-del mice compared to WT controls during the dark phase. We reproduced these findings by our discoveries of reduced percent time in paradoxical sleep using 2-h time bins and counts/10 s of paradoxical sleep. In contrast, we observed these deficits across both light and dark cycles. Methodologies and coding algorithms may account for the differences. We are continuing to examine this discrepancy. This earlier work also described shorter NREM bout durations throughout the dark cycle in *Ube3a*-del mice, and specifically NREM frequency of bouts was shorter at the beginning of the dark cycle and then increased over time. The number of NREM bouts also remained high during the early dark in *Ube3a*-del mice. These results concluded that *Ube3a*-del mice had a “fragmented” NREM sleep in the dark cycle compared to WT mice. While we did not observe changes in percent slow-wave sleep time or count across our 24-h sampling window, we did observe trends toward reduced slow-wave sleep time across the total recording. Furthermore, *Ube3a-*del mice showed impairments in total sleep time, namely through reductions in mean paradoxical sleep time and trends toward reduced slow-wave sleep compared to WT animals. *Ube3a-*del mice took longer to reach paradoxical sleep compared to WT, suggesting difficulty reaching deep sleep stages and, once there, difficulty remaining in those stages. We believe our data corroborate the impaired sleep phenotype described by Ehlen and Philpot although our signal collection methods and output metrics differed.

For the first time, in a preclinical model of AS, we have defined sleep spindles with custom-built automation, following manually filtering data and processed via our custom algorithm. Sleep spindles are thalamocortical oscillations ranging from 11 to 16 Hz and are theoretically thought to mediate memory consolidation. There have been reports of sleep spindle deficits in the AS population, but not in a mouse model of AS [43]. *Ube3a-*del mice exhibited less spindles, as we hypothesized given the reduction in sleep spindles observed clinically in AS [43]. Interestingly, when accounting for total sleep time, spindle count was significantly higher in *Ube3a*-del mice compared to WT, but not directly correlated with total sleep time, mean time in paradoxical sleep and mean time in slow-wave sleep. From these data we posit that spindle production is abnormal in *Ube3a*-del as observed by reduced total amount of spindles and increased spindle frequency during shorter sleep periods. Future studies will be required to investigate the nuances of these phenotypes.

Fewer, abnormal sleep spindles have been historically implicated in intellectual disabilities [55, 56] and for numerous genetic NDDs, such as Phelan–McDermid and Prader–Willi syndromes [57, 58] as well as childhood epilepsies [59] 60 and ASDs [61, 62]. Sleep spindles have been reported altered when mGluR5 is deficient [63], and mGluR5 dysregulation has been postulated as underlying Fragile X Syndrome phenotypes, another NDD with ASD and poor sleep regulation [64, 65]. Interestingly, there was a significant negative correlation between spindles and delta power, where animals with higher delta power tended to have lower spindle counts. It is unclear whether elevated delta power directly correlates with any core clinical features of AS, but these data offer a promising link between delta rhythmicity and possible cognitive outcome measures.

Limitations to this work were our lack of inclusion of anatomical substrate analysis either by gross MRI scan or histopathology in regions to identify neuronal integrity or lack thereof. While less regionally specific than typical depth recording techniques, we sought to use a more clinically analogous EEG recording method that collected signals from the surface of the skull and was wireless to allow for free movement of test animals. We hypothesized that quantifying EEG measures in subjects’ home cage environment using wireless telemetry devices would be sufficient to assess seizures, sleep deficits and spectral band abnormalities associated with models of AS and NDDs as well as offer translational observation of global neuronal activity, as previously described [44]. Further limitations were our lack of correlation of the reduction of spindles with lower cognitive behavior observed by others in AS mice, a popular theory of spindle function [55, 56].

In summary, our data enhance rigor and translatability of EEG readouts as having biomarker potential for preclinical testing of therapeutics. Our study provides important corroboration of previous reports of rodent epileptiform with EEG that illustrated that *Ube3a*-del mice had increased epileptiform spiking activity and elevated delta power, which corroborates reported work and recapitulates clinical reports in AS. This is the first report to use a cortical surface-based recording by a wireless telemetry device over tethered/fixed head-mount depth recordings. Less time in both paradoxical and slow-wave sleep, longer latencies to paradoxical sleep stages and total less sleep time in *Ube3a*-del mice were observed compared to WT. For the first time, we detected fewer sleep spindles in the AS mouse model, a critical marker of memory consolidation during sleep, in a preclinical model of AS. This study was limited to the exon 2 deletion mouse model, and future work will investigate the rat model of AS, containing a complete Ube3a deletion and pair EEG with behavior. Our data enhance rigor and translatability of EEG, spectral power and sleep as beneficial outcomes for therapeutic testing.

## Supplementary Information

**Additional file 1: Supplemental Figure 1**.* Ube3a*-del mice exhibited similar latencies to loss of righting and death after convulsant administration. Seizure susceptibility measures were observed for 30 min after an i.p. injection of 80 mg/kg PTZ. While reduced latencies to both first jerk and generalized clonictonic seizures were observed in* Ube3a*-del mice Figure 1 (**a**, **b**), no genotype differences were detected in either **A** latency to loss of righting or **B** death. *p < 0.05, Student’s t-test between genotype.

**Additional file 1: Supplemental Figure 2**. No significant correlations were detected across spiking events and delta rhythmicity, sleep metrics and delta rhythmicity, or spiking and sleep in* Ube3a*-del mice. Spiking events and delta power were correlated in **A** Cohort 3* Ube3a*-del mice and **B** Cohort 2* Ube3a*-del mice, with no significant relationship detected. Similarly, when analyzing **C**–**F** mean time in paradoxical sleep, mean time in slow-wave sleep, latency to paradoxical sleep and total sleep time with delta power and **G**–**J** mean time in paradoxical sleep, mean time in slow-wave sleep, latency to paradoxical sleep and total sleep time with spiking events in Cohort 3 animals, no significant correlations were found. *p < 0.05, linear regression for correlation across behaviors.

## Data Availability

Data supporting our findings can be found in the UC Davis School of medicine Shared Network. Please contact author for data/custom algorithm requests.

## References

[1] Kishino T, Lalande M, Wagstaff J (1997). UBE3A/E6-AP mutations cause Angelman syndrome. Nat Genet.

[2] Chamberlain SJ, Lalande M (2010). Angelman syndrome, a genomic imprinting disorder of the brain. J Neurosci.

[3] Williams CA, Beaudet AL, Clayton-Smith J, Knoll JH, Kyllerman M, Laan LA, Magenis RE, Moncla A, Schinzel AA, Summers JA, Wagstaff J (2006). Angelman syndrome 2005: updated consensus for diagnostic criteria. Am J Med Genet A.

[4] Minassian BA, DeLorey TM, Olsen RW, Philippart M, Bronstein Y, Zhang Q, Guerrini R, Van Ness P, Livet MO, Delgado-Escueta AV (1998). Angelman syndrome: correlations between epilepsy phenotypes and genotypes. Ann Neurol.

[5] Thibert RL, Conant KD, Braun EK, Bruno P, Said RR, Nespeca MP, Thiele EA (2009). Epilepsy in Angelman syndrome: a questionnaire-based assessment of the natural history and current treatment options. Epilepsia.

[6] Uemura N, Matsumoto A, Nakamura M, Watanabe K, Negoro T, Kumagai T, Miura K, Ohki T, Mizuno S, Okumura A (2005). Evolution of seizures and electroencephalographical findings in 23 cases of deletion type Angelman syndrome. Brain Dev.

[7] Khan N, Cabo R, Tan WH, Tayag R, Bird LM (2019). Healthcare burden among individuals with Angelman syndrome: findings from the Angelman Syndrome Natural History Study. Mol Genet Genom Med.

[8] Boyd SG, Harden A, Patton MA (1988). The EEG in early diagnosis of the Angelman (happy puppet) syndrome. Eur J Pediatr.

[9] Leyser M, Penna PS, de Almeida AC, Vasconcelos MM, Nascimento OJ (2014). Revisiting epilepsy and the electroencephalogram patterns in Angelman syndrome. Neurol Sci.

[10] Goldman SE, Bichell TJ, Surdyka K, Malow BA (2012). Sleep in children and adolescents with Angelman syndrome: association with parent sleep and stress. J Intellect Disabil Res.

[11] Jiang YH, Armstrong D, Albrecht U, Atkins CM, Noebels JL, Eichele G, Sweatt JD, Beaudet AL (1998). Mutation of the Angelman ubiquitin ligase in mice causes increased cytoplasmic p53 and deficits of contextual learning and long-term potentiation. Neuron.

[12] Nawaz Z, Lonard DM, Smith CL, Lev-Lehman E, Tsai SY, Tsai MJ, O'Malley BW (1999). The Angelman syndrome-associated protein, E6-AP, is a coactivator for the nuclear hormone receptor superfamily. Mol Cell Biol.

[13] Wallace ML, Burette AC, Weinberg RJ, Philpot BD (2012). Maternal loss of Ube3a produces an excitatory/inhibitory imbalance through neuron type-specific synaptic defects. Neuron.

[14] Roden WH, Peugh LD, Jansen LA (2010). Altered GABA(A) receptor subunit expression and pharmacology in human Angelman syndrome cortex. Neurosci Lett.

[15] Judson MC, Wallace ML, Sidorov MS, Burette AC, Gu B, van Woerden GM, King IF, Han JE, Zylka MJ, Elgersma Y (2016). GABAergic neuron-specific loss of Ube3a causes angelman syndrome-like EEG abnormalities and enhances seizure susceptibility. Neuron.

[16] Berg EL, Pride MC, Petkova SP, Lee RD, Copping NA, Shen Y, Adhikari A, Fenton TA, Pedersen LR, Noakes LS (2020). Translational outcomes in a full gene deletion of ubiquitin protein ligase E3A rat model of Angelman syndrome. Transl Psychiatry.

[17] Dodge A, Peters MM, Greene HE, Dietrick C, Botelho R, Chung D, Willman J, Nenninger AW, Ciarlone S, Kamath SG (2020). Generation of a novel rat model of angelman syndrome with a complete Ube3a gene deletion. Autism Res.

[18] Adhikari A, Copping NA, Onaga B, Pride MC, Coulson RL, Yang M, Yasui DH, LaSalle JM, Silverman JL (2018). Cognitive deficits in the Snord116 deletion mouse model for prader-willi syndrome. Neurobiol Learn Mem.

[19] Bozdagi O, Sakurai T, Papapetrou D, Wang X, Dickstein DL, Takahashi N, Kajiwara Y, Yang M, Katz AM, Scattoni ML (2010). Haploinsufficiency of the autism-associated Shank3 gene leads to deficits in synaptic function, social interaction, and social communication. Mol Autism.

[20] Brielmaier J, Matteson PG, Silverman JL, Senerth JM, Kelly S, Genestine M, Millonig JH, DiCicco-Bloom E, Crawley JN (2012). Autism-relevant social abnormalities and cognitive deficits in engrailed-2 knockout mice. PLoS ONE.

[21] Copping NA, Berg EL, Foley GM, Schaffler MD, Onaga BL, Buscher N, Silverman JL, Yang M (2016). Touchscreen learning deficits and normal social approach behavior in the Shank3B model of Phelan-McDermid Syndrome and autism. Neuroscience.

[22] Copping NA, Christian SGB, Ritter DJ, Islam MS, Buscher N, Zolkowska D, Pride MC, Berg EL, LaSalle JM, Ellegood J (2017). Neuronal overexpression of Ube3a isoform 2 causes behavioral impairments and neuroanatomical pathology relevant to 15q11.2–q13.3 duplication syndrome. Hum Mol Genet.

[23] Gompers AL, Su-Feher L, Ellegood J, Copping NA, Riyadh MA, Stradleigh TW, Pride MC, Schaffler MD, Wade AA, Catta-Preta R (2017). Germline Chd8 haploinsufficiency alters brain development in mouse. Nat Neurosci.

[24] Kelly E, Schaeffer SM, Dhamne SC, Lipton JO, Lindemann L, Honer M, Jaeschke G, Super CE, Lammers SH, Modi ME (2018). mGluR5 modulation of behavioral and epileptic phenotypes in a mouse model of tuberous sclerosis complex. Neuropsychopharmacology.

[25] Wohr M, Silverman JL, Scattoni ML, Turner SM, Harris MJ, Saxena R, Crawley JN (2013). Developmental delays and reduced pup ultrasonic vocalizations but normal sociability in mice lacking the postsynaptic cell adhesion protein neuroligin2. Behav Brain Res.

[26] Yang M, Bozdagi O, Scattoni ML, Wohr M, Roullet FI, Katz AM, Abrams DN, Kalikhman D, Simon H, Woldeyohannes L (2012). Reduced excitatory neurotransmission and mild autism-relevant phenotypes in adolescent Shank3 null mutant mice. J Neurosci.

[27] Chung L, Bey AL, Towers AJ, Cao X, Kim IH, Jiang YH (2018). Lovastatin suppresses hyperexcitability and seizure in Angelman syndrome model. Neurobiol Dis.

[28] Ciarlone SL, Grieco JC, D'Agostino DP, Weeber EJ (2016). Ketone ester supplementation attenuates seizure activity, and improves behavior and hippocampal synaptic plasticity in an Angelman syndrome mouse model. Neurobiol Dis.

[29] Ciarlone SL, Wang X, Rogawski MA, Weeber EJ (2016). Effects of the synthetic neurosteroid ganaxolone on seizure activity and behavioral deficits in an Angelman syndrome mouse model. Neuropharmacology.

[30] Mandel-Brehm C, Salogiannis J, Dhamne SC, Rotenberg A, Greenberg ME (2015). Seizure-like activity in a juvenile Angelman syndrome mouse model is attenuated by reducing arc expression. Proc Natl Acad Sci U S A.

[31] Sidorov MS, Deck GM, Dolatshahi M, Thibert RL, Bird LM, Chu CJ, Philpot BD (2017). Delta rhythmicity is a reliable EEG biomarker in Angelman syndrome: a parallel mouse and human analysis. J Neurodev Disord.

[32] Born HA, Dao AT, Levine AT, Lee WL, Mehta NM, Mehra S, Weeber EJ, Anderson AE (2017). Strain-dependence of the Angelman Syndrome phenotypes in Ube3a maternal deficiency mice. Sci Rep.

[33] Gu B, Zhu M, Glass MR, Rougie M, Nikolova VD, Moy SS, Carney PR, Philpot BD (2019). Cannabidiol attenuates seizures and EEG abnormalities in Angelman syndrome model mice. J Clin Investig.

[34] Copping NA, Adhikari A, Petkova SP, Silverman JL (2019). Genetic backgrounds have unique seizure response profiles and behavioral outcomes following convulsant administration. Epilepsy Behav.

[35] Uygun DS, Katsuki F, Bolortuya Y, Aguilar DD, McKenna JT, Thankachan S, McCarley RW, Basheer R, Brown RE, Strecker RE, McNally JM (2019). Validation of an automated sleep spindle detection method for mouse electroencephalography. Sleep.

[36] Kim D, Hwang E, Lee M, Sung H, Choi JH (2015). Characterization of topographically specific sleep spindles in mice. Sleep.

[37] Thibert RL, Larson AM, Hsieh DT, Raby AR, Thiele EA (2013). Neurologic manifestations of Angelman syndrome. Pediatr Neurol.

[38] Rubin DI, Patterson MC, Westmoreland BF, Klass DW (1997). Angelman’s syndrome: clinical and electroencephalographic findings. Electroencephalogr Clin Neurophysiol.

[39] Meng L, Person RE, Huang W, Zhu PJ, Costa-Mattioli M, Beaudet AL (2013). Truncation of Ube3a-ATS unsilences paternal Ube3a and ameliorates behavioral defects in the Angelman syndrome mouse model. PLoS Genet.

[40] Daily JL, Nash K, Jinwal U, Golde T, Rogers J, Peters MM, Burdine RD, Dickey C, Banko JL, Weeber EJ (2011). Adeno-associated virus-mediated rescue of the cognitive defects in a mouse model for Angelman syndrome. PLoS ONE.

[41] Bailus BJ, Pyles B, McAlister MM, O'Geen H, Lockwood SH, Adams AN, Nguyen JT, Yu A, Berman RF, Segal DJ (2016). Protein delivery of an artificial transcription factor restores widespread Ube3a expression in an Angelman syndrome mouse brain. Mol Ther.

[42] Huang HS, Burns AJ, Nonneman RJ, Baker LK, Riddick NV, Nikolova VD, Riday TT, Yashiro K, Philpot BD, Moy SS (2013). Behavioral deficits in an Angelman syndrome model: effects of genetic background and age. Behav Brain Res.

[43] den Bakker H, Sidorov MS, Fan Z, Lee DJ, Bird LM, Chu CJ, Philpot BD (2018). Abnormal coherence and sleep composition in children with Angelman syndrome: a retrospective EEG study. Mol Autism.

[44] Dhamne SC, Silverman JL, Super CE, Lammers SHT, Hameed MQ, Modi ME, Copping NA, Pride MC, Smith DG, Rotenberg A (2017). Replicable in vivo physiological and behavioral phenotypes of the Shank3B null mutant mouse model of autism. Mol Autism.

[45] Ehlen JC, Jones KA, Pinckney L, Gray CL, Burette S, Weinberg RJ, Evans JA, Brager AJ, Zylka MJ, Paul KN (2015). Maternal Ube3a loss disrupts sleep homeostasis but leaves circadian rhythmicity largely intact. J Neurosci.

[46] Frohlich J, Miller MT, Bird LM, Garces P, Purtell H, Hoener MC, Philpot BD, Sidorov MS, Tan WH, Hernandez MC (2019). Electrophysiological phenotype in Angelman syndrome differs between genotypes. Biol Psychiatry.

[47] Finucane BM, Lusk L, Arkilo D, Chamberlain S, Devinsky O, Dindot S, Jeste SS, LaSalle JM, Reiter LT, Schanen NC (2016). 15q duplication syndrome and related disorders.

[48] Frohlich J, Senturk D, Saravanapandian V, Golshani P, Reiter LT, Sankar R, Thibert RL, DiStefano C, Huberty S, Cook EH, Jeste SS (2016). A quantitative electrophysiological biomarker of duplication 15q11.2–q13.1 syndrome. PLoS ONE.

[49] Jeste SS, Geschwind DH (2014). Disentangling the heterogeneity of autism spectrum disorder through genetic findings. Nat Rev Neurol.

[50] Didden R, Korzilius H, Smits MG, Curfs LM (2004). Sleep problems in individuals with Angelman syndrome. Am J Ment Retard.

[51] Miano S, Bruni O, Leuzzi V, Elia M, Verrillo E, Ferri R (2004). Sleep polygraphy in Angelman syndrome. Clin Neurophysiol.

[52] Walz NC, Beebe D, Byars K (2005). Sleep in individuals with Angelman syndrome: parent perceptions of patterns and problems. Am J Ment Retard.

[53] Bruni O, Ferri R, D'Agostino G, Miano S, Roccella M, Elia M (2004). Sleep disturbances in Angelman syndrome: a questionnaire study. Brain Dev.

[54] Trickett J, Heald M, Oliver C (2017). Sleep in children with Angelman syndrome: parental concerns and priorities. Res Dev Disabil.

[55] Gibbs EL, Gibbs FA (1962). Extreme spindles: correlation of electroencephalographic sleep pattern with mental retardation. Science.

[56] Rohde JA, Kooi KA, Richey ET (1969). Sleep spindles, mental retardation and epilepsy. Electroencephalogr Clin Neurophysiol.

[57] Ingiosi AM, Schoch H, Wintler T, Singletary KG, Righelli D, Roser LG, Medina E, Risso D, Frank MG, Peixoto L (2019). Shank3 modulates sleep and expression of circadian transcription factors. Elife.

[58] Dykens EM, Lee E, Roof E (2011). Prader-Willi syndrome and autism spectrum disorders: an evolving story. J Neurodev Disord.

[59] Lesca G, Rudolf G, Labalme A, Hirsch E, Arzimanoglou A, Genton P, Motte J, de Saint MA, Valenti MP, Boulay C (2012). Epileptic encephalopathies of the Landau-Kleffner and continuous spike and waves during slow-wave sleep types: genomic dissection makes the link with autism. Epilepsia.

[60] Arican P, Gencpinar P, Olgac Dundar N (2019). A new cause of developmental and epileptic encephalopathy with continuous spike-and-wave during sleep: CDKL5 disorder. Neurocase.

[61] Bourgeron T (2007). The possible interplay of synaptic and clock genes in autism spectrum disorders. Cold Spring Harb Symp Quant Biol.

[62] Farmer CA, Chilakamarri P, Thurm AE, Swedo SE, Holmes GL, Buckley AW (2018). Spindle activity in young children with autism, developmental delay, or typical development. Neurology.

[63] Aguilar DD, Strecker RE, Basheer R, McNally JM (2020). Alterations in sleep, sleep spindle, and EEG power in mGluR5 knockout mice. J Neurophysiol.

[64] Boone CE, Davoudi H, Harrold JB, Foster DJ (2018). Abnormal sleep architecture and hippocampal circuit dysfunction in a mouse model of fragile X syndrome. Neuroscience.

[65] Sare RM, Harkless L, Levine M, Torossian A, Sheeler CA, Smith CB (2017). Deficient sleep in mouse models of fragile X syndrome. Front Mol Neurosci.

