# Supplementary figures and images for: Abnormal electrophysiological phenotypes and sleep deficits in a mouse model of Angelman Syndrome
Source: Mol Autism. 2021 Feb 6;12:9. doi: 10.1186/s13229-021-00416-y (PMC7866697; doi:10.1186/s13229-021-00416-y)

**a**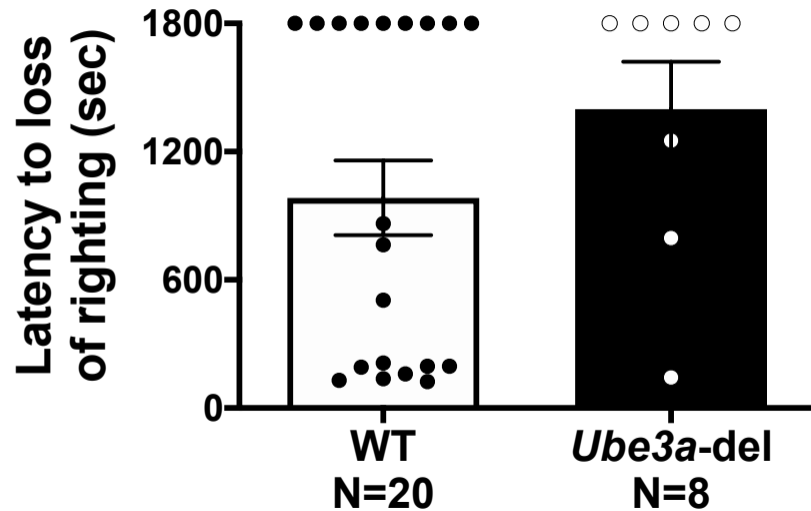**b**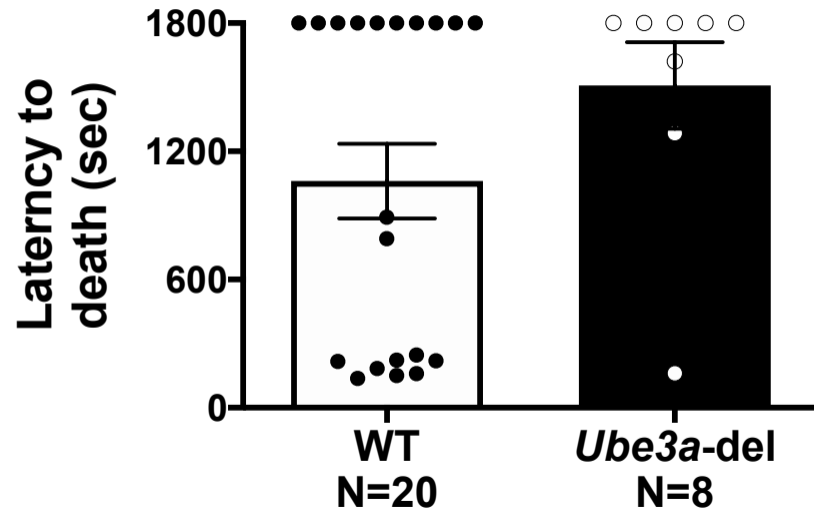

Supplement: Supplementary file 1 — Additional file 1: Supplemental Figure 1. Ube3a-del mice exhibited similar latencies to loss of righting and death after convulsant administration. Seizure susceptibility measures were observed for 30 min after an i.p. injection of 80 mg/kg PTZ. While reduced latencies to both first jerk and generalized clonictonic seizures were observed in Ube3a-del mice Figure 1 (a, b), no genotype differences were detected in either A latency to loss of righting or B death. *p < 0.05, Student’s t-test between genotype. [file 13229_2021_416_MOESM1_ESM.pdf]

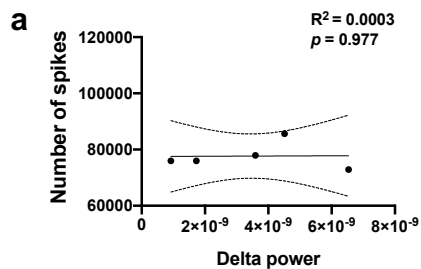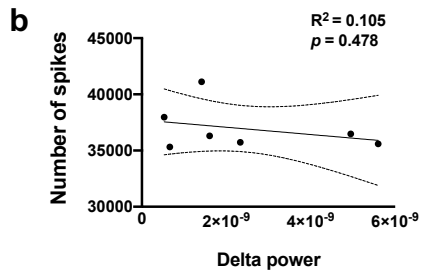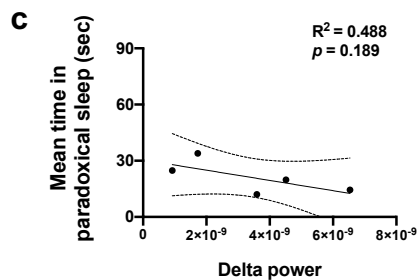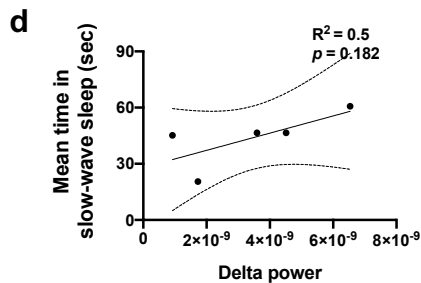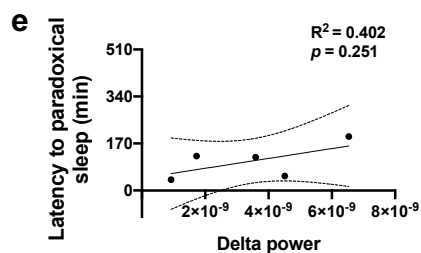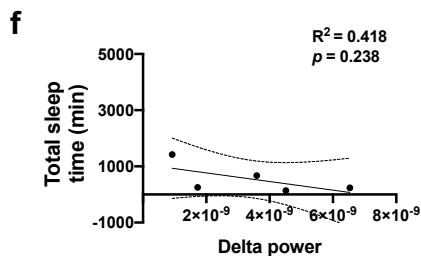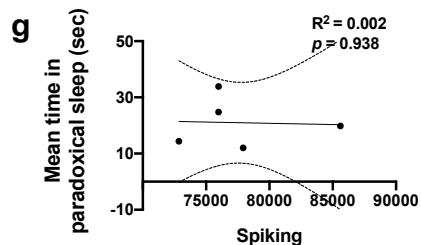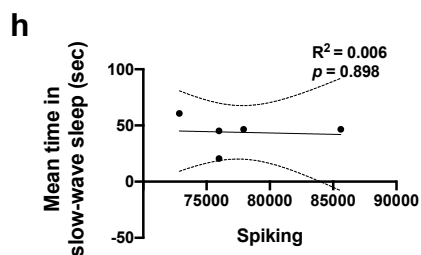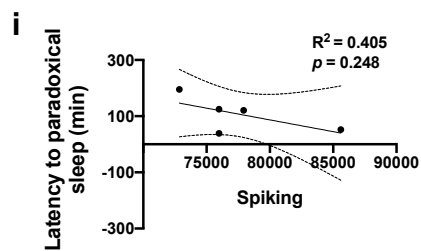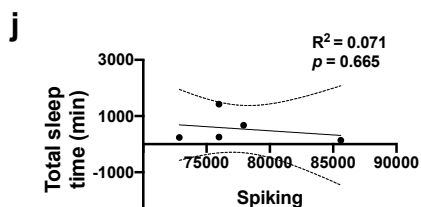

Supplement: Supplementary file 2 — Additional file 1: Supplemental Figure 2. No significant correlations were detected across spiking events and delta rhythmicity, sleep metrics and delta rhythmicity, or spiking and sleep in Ube3a-del mice. Spiking events and delta power were correlated in A Cohort 3 Ube3a-del mice and B Cohort 2 Ube3a-del mice, with no significant relationship detected. Similarly, when analyzing C–F mean time in paradoxical sleep, mean time in slow-wave sleep, latency to paradoxical sleep and total sleep time with delta power and G–J mean time in paradoxical sleep, mean time in slow-wave sleep, latency to paradoxical sleep and total sleep time with spiking events in Cohort 3 animals, no significant correlations were found. *p < 0.05, linear regression for correlation across behaviors. [file 13229_2021_416_MOESM2_ESM.pdf]
